# Major Effect of Oxidative Stress on the Male, but Not Female, SP-A1 Type II Cell miRNome

**DOI:** 10.3389/fimmu.2019.01514

**Published:** 2019-07-10

**Authors:** George T. Noutsios, Nithyananda Thorenoor, Xuesheng Zhang, David S. Phelps, Todd M. Umstead, Faryal Durrani, Joanna Floros

**Affiliations:** ^1^Center for Host Defense, Inflammation, and Lung Disease (CHILD) Research, Department of Pediatrics, College of Medicine, Pennsylvania State University, Hershey, PA, United States; ^2^Department of Obstetrics and Gynecology, College of Medicine, Pennsylvania State University, Hershey, PA, United States

**Keywords:** alveolar epithelium, surfactant protein A, ozone, sex differences, *MAPK*

## Abstract

Pulmonary surfactant protein A (SP-A) plays an important role in surfactant metabolism and lung innate immunity. In humans there are two proteins, SP-A1 and SP-A2, encoded by *SFTPA1* and *SFTPA2*, respectively, which are produced by the alveolar type II cells (T2C). We sought to investigate the differential influence of SP-A1 and SP-A2 in T2C miRNome under oxidative stress (OxS). SP-A knock out (KO) and hTG male and female mice expressing SP-A1 or SP-A2 as well as gonadectomized (Gx) mice were exposed to O_3_-induced oxidative stress (OxS) or filtered air (FA). Expression of miRNAs and mRNAs was measured in the T2C of experimental animals. (a) In SP-A1 males after normalizing to KO males, significant changes were observed in the miRNome in terms of sex-OxS effects, with 24 miRNAs being differentially expressed under OxS. (b) The mRNA targets of the dysregulated miRNAs included *Ago2, Ddx20, Plcg2, Irs1, Elf2, Jak2, Map2k4, Bcl2, Ccnd1*, and *Vhl*. We validated the expression levels of these transcripts, and observed that the mRNA levels of all of these targets were unaffected in SP-A1 T2C but six of these were significantly upregulated in the KO (except *Bcl2* that was downregulated). (c) Gondadectomy had a major effect on the expression of miRNAs and in three of the mRNA targets (*Irs1, Bcl2*, and *Vhl*). *Ccnd1* was upregulated in KO regardless of Gx. (d) The targets of the significantly changed miRNAs are involved in several pathways including *MAPK* signaling pathway, cell cycle, anti-apoptosis, and other. In conclusion, in response to OxS, SP-A1 and male hormones appear to have a major effect in the T2C miRNome.

## Introduction

Ambient ozone (O_3_)-induced oxidative stress (OxS) is one of the major environmental factors contributing to the occurrence and development of upper and lower airway disease, including chronic rhinosinusitis (CRS) (1), asthma, and chronic obstructive lung disease (COPD) (2, 3). In the distal lung, the alveolar epithelial cells provide the first line of defense against environmental pathogens such as O_3_, pollutants, bacteria, viruses, and allergens by producing a number of protective factors (4). In addition to secreting pulmonary surfactant to reduce the alveolar surface tension, they produce chemokines and cytokines that regulate alveolar inflammatory responses as well as proteinases and proteinase inhibitors (5). Upon environmental stress such as that of OxS, O_3_ increases the production of reactive oxygen species (ROS) (6) that disrupt the alveolar epithelial cell barrier function by the dissociation of the tight junctions of alveolar epithelium (7), thereby allowing the entrance of opportunistic bacteria such as *Pseudomonas aeruginosa* to infect the alveolar epithelium. Exposure to O_3_ also reduces pulmonary surfactant secretion (8). The alveolar epithelium initiates a self-repair process by recruitment, proliferation, and differentiation of new epithelial cells to maintain the structural and functional traits that are required to maintain a normal respiratory function (9).

The alveolar epithelium is comprised of two different cells types, the alveolar type I cells (T1C) and the type II cells (T2C) that are in close proximity with the alveolar macrophages (AM) that reside in the alveolar space. The T1C are large, flat cells with a thin attenuated cytoplasm that line 90% of the alveolar surface. This distinct shape enables them to facilitate O_2_/CO_2_ gas exchange by minimizing the diffusion distance between the alveolar surface and the blood (10). The T2C cover ~10% of the alveolar surface and their main function is to produce and secrete pulmonary surfactant, a phospholipid and protein mixture, which lowers the surface tension in the alveolus during the respiration process. T2C possess unique secretory organelles, called lamellar bodies, which contain surfactant lipids and surfactant proteins A (SP-A), SP-B, and SP-C. Also, T2C play a very important role in the epithelium repair process after lung injury and are considered the progenitor cells of the alveolar epithelium. Upon epithelial damage T2C proliferate as new T2C and they differentiate into T1C repairing the scarred epithelial surface (9, 11, 12).

SP-A is the most abundant protein in pulmonary surfactant and has both surfactant-related functions and innate immunity functions (13–17). SP-A knock-out mouse studies have revealed important host defense functions of SP-A, where KO mice are more vulnerable to bacterial infections compared to the mice that express SP-A (18–22). Also, SP-A has been shown to have regulatory effects on the proteome, function, cell shape, and activation state of AM (21, 23–26). OxS stress increases the production of reactive oxygen species (ROS) (6) and these in turn damage the alveolar epithelium (27), oxidize SP-A, and compromise innate immune functions (23, 28–33). In humans however, unlike in rodents, there are two different genes, *SFTPA1* and *SFTPA2*, encoding SP-A1 and SP-A2 proteins, respectively. We have previously shown that SP-A1 and SP-A2 differentially affect the proteomic expression in AM (34, 35), the AM function (36–38), surfactant secretion (39), structure of surfactant monolayers (40, 41) and more recently we have shown that SP-A1 and SP-A2 differentially regulate the AM miRNome and antioxidant pathways in the AM (42), lung function mechanics (43), and survival after *K. pneumoniae* infection (44). MicroRNAs (miRNAs) have also been shown to differentially affect SP-A1 and SP-A2 expression (45), and also contribute to the maintenance of the T2C phenotype (46).

In the present study, using humanized transgenic (hTG) mice, where each expresses either SP-A1 or SP-A2, we sought to investigate the differential influence of SP-A1 and SP-A2 on the T2C miRNome under the effect of OxS. We found that the T2C miRNome is regulated in response to OxS and that O_3_ exposure has a major effect on the male SP-A1 miRNome. We also show that sex hormones play a role in T2C miRNome under the studied conditions.

## Methods

### Oxidative Stress Animal Model

Twelve weeks old humanized transgenic (hTG) C57BL/6 mice (males and females) each carrying human SP-A1 (6A^2^), SP-A2 (1A^0^) (47), as well as SP-A knock-out (KO) were used in the present study. Females were synchronized for 7 days as described previously (42) to stimulate estrus. A total of *n* = 52 mice (36 for miRNA study, 16 for gonadectomy analysis). Protocols involving animal procedures were approved by the Institutional Animal Care and Use Committee at the Pennsylvania State University College of Medicine.

**Figure 1:**
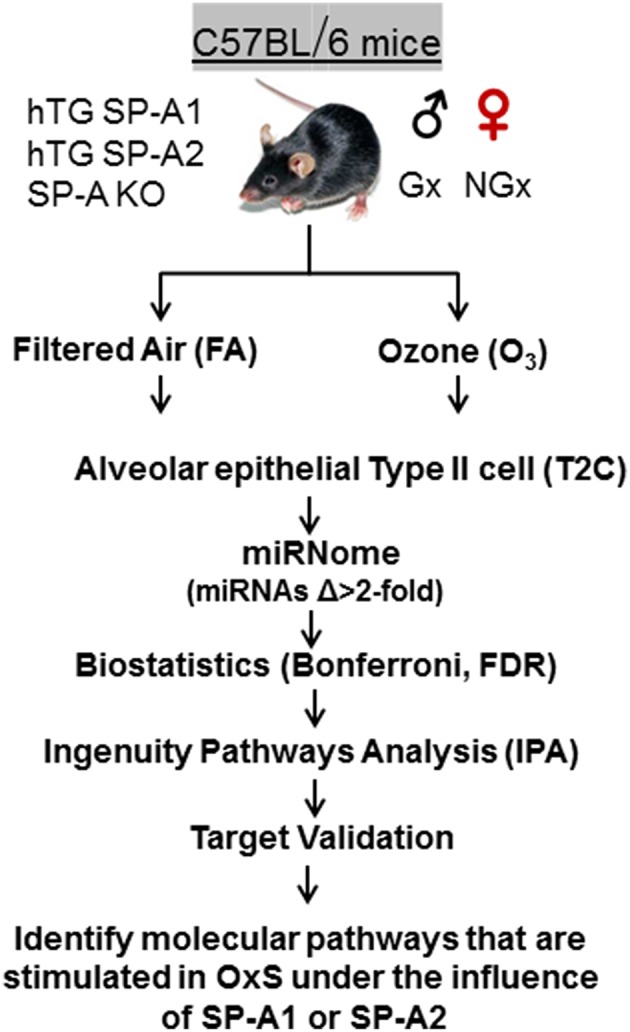
Experimental workflow of the present study. hTG, humanized transgenic that each carries a single SP-A1 (6A^2^) or SP-A2 (1A^0^) variant; SP-A-KO, knock out; Gx, gonadectomized; NGx, non-gonadectomized; FA, filter air; O_3_, ozone; T2C, Alveolar epithelial Type II cells; Δ, difference; FDR, false discovery rate; IPA, ingenuity pathway analysis; OxS, oxidative stress.

Animals were exposed to 2 ppm ozone (O_3_) or filtered air (FA) (control) at 25°C as described previously (42, 48). We used 3 mice per group. i.e., 3 males, 3 females, 3 SP-A KO, 3 hTG SP-A2, 3 hTG SP-A1, 3 for O_3_, and FA exposure (*n* = 36). All O_3_ and FA exposures were conducted in parallel as described (49). Mice were sacrificed 4 h post exposure. Each animal was analyzed individually, and we did not pool any samples. Summary of the experimental workflow is depicted in Figure 1.

### Mouse Alveolar Type II Cells Isolation

Mouse type II cells were isolated based on a modified method that was described previously (50). Briefly, mice were anesthetized with intraperitoneal injection of 87.5 mg/kg ketamine and 12.5 mg/kg xylazine and exsanguinated by cutting the inferior vena cava. Cardiac perfusion of the lung was performed with 10 mL of normal saline solution followed by endotracheal intubation and infusion of the lungs with 3 mL solution of 50 U/ml dispase II (Sigma-Aldrich, St. Louis, MO) in HBSS 1x and sealed with 0.5 mL of 1% solution of low melting agarose (Sigma-Aldrich). The lungs were removed from the thoracic cavity and lung lobes digested in 15 mL tube containing 2 mL of dispase II for 45 min at 37°C with constant shaking at 150 rpm. Digested lungs were dissected and homogenized in 7 mL of complete DMEM solution, supplemented with 10 μL DNase I (5,000 Kunitz U/ml Sigma-Aldrich). The lung epithelial cells were filtered through a 100 and 40 μm strainer, passed through 20 μm- nylon mesh, cells were collected by centrifugation at 130 xg for 8 min, and resuspended in 10 mL of DMEM/25 mM HEPES/10% FBS/1x AB/AM. Negative selection of T2C was performed by incubating the cell suspensions in 10-cm cultured dishes coated with 42 μg anti-mouse CD45 (targeting hematopoietic cells) and 16 μg anti-mouse CD16/32 (BD Pharmingen, San Jose, CA) (targeting alveolar macrophages) at 37°C, 10% CO_2_ for 2 h. Non-attached cells were centrifuged, washed with 1x PBS (Gibco, Waltham, MA) and counted. A fraction was used to prepare cytospins, cells were stained, and a differential cell count was performed. T2C purity was 95% as assessed by Papanikolaou staining. The remaining T2C pellet was resuspended in 500 μL solution of DMEM supplemented with 40% fetal bovine serum (Gibco) and 10% DMSO (Sigma Aldrich, St. Louis, MO) and T2C were cryopreserved in liquid nitrogen until further use.

### Gonadectomy and Ozone Exposure

Male and female SP-A1 and KO mice were gonadectomized (Gx) and exposed to O_3_ (2 ppm) for 3 h and were sacrificed 4 h post OxS as described (51). The differentially expressed miRNAs from Gx samples were identified by RNA sequencing as described previously (42, 52). The miRNAs identified from Gx mice were selected for analysis and changes in miRNA expression in SP-A1 mice were calculated by normalizing to KO as described previously (42). Samples from 16 animals (8 males and 8 females for SP-A1 and KO) were individually analyzed.

### Isolation of miRNAs, qRT-PCR, and Statistical Analysis

Total RNA from the isolated mouse T2C was prepared using QIAzol Lysis Reagent (Qiagen, Valencia, CA) and the miRNA-enriched fraction was purified and used to generate cDNA, and then served as a template for real-time qPCR. Expression profiles of the 372 most abundantly expressed and best-characterized miRNAs in miRBase were then studied as described previously (42). The expression of miRNAs from FA and O_3_ exposed T2C samples from SP-A1, SP-A2, and KO mice were analyzed as described previously (42). The variability across the 3 samples was assessed by *p*-values (*p* < 0.0166) and miRNAs with significantly changed levels were studied further (*p* < 0.0166). Bonferroni correction applied for sex, treatment, and genotype variability. The miRNA:gene target interactions were identified and reported in a format which enables direct transfer of results to genomic databases cataloging validated miRNA-target interactions as described previously (53, 54).

### Gene Expression Analysis

To assess the expression of levels of *Ago2, Ddx20, Plcg2, Irs1, Elf2, Bcl2, Jak2, Map2k4, Bcl2, Ccnd1*, and *Vhl* genes at mRNA level in the male non-gonadectomized (NGx) and gonadectomized (Gx) KO and SP-A1 T2C, we performed qRT-PCR as described previously (42). The specific RT2 qPCR Primer assay was purchased from Qiagen. Cell samples were obtained from 3 separate animals/treatment (FA and O_3_), and each sample was analyzed in triplicate/animal and quantified relative to *Gapdh* mRNA expression.

## Results

### SP-A1 and SP-A2 Differentially Regulate the T2C miRNome

**Figure 2:**
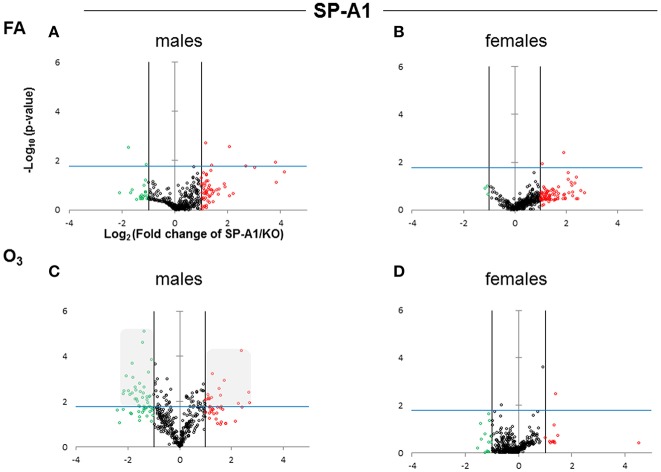
Volcano plots indicating the statistical significance of SP-A1 T2C miRNome expression levels compared to SP-A KO under FA or O_3_ exposure for males and females. The x-axis plots the log_2_ of the fold-changes, while the y-axis plots the –log_10_ of their *p*-values based on *t*-test of the replicate raw Ct data (section Materials and Methods). Each plot has three vertical lines. The middle vertical line that is graded corresponds to zero changes. The lines on either side represent ≥2-fold differences. Dots in the volcano plots above the blue horizontal line identify fold-changes with statistical significance of at least the Bonferroni corrected *p* < 0.0166. The red and green dots represent miRNAs that were upregulated ≥2 fold and downregulated ≥2 fold, respectively, compared to KO. Black dots signify miRNAs that were regulated <2-fold times (i.e., x ≤ 2, x is the fold change). **(A)** Male SP-A1 mice compared to KO exposed to FA; **(B)** Female SP-A1 mice compared to KO exposed to FA; **(C)** Male SP-A1 mice compared to KO after O_3_ exposure; **(D)** Female SP-A1 mice compared to KO after O_3_ exposure. The shaded gray area in **(C)** shows show the differences in the miRNAs that are highly and significantly regulated.

**Figure 3:**
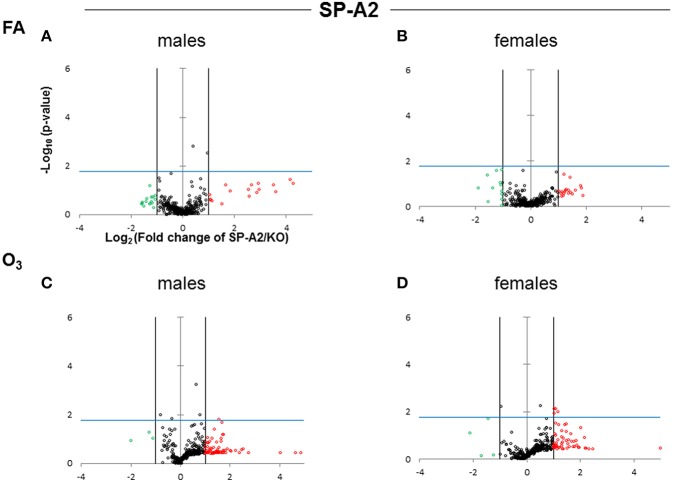
Volcano plots (as described in Figure 2) indicating the statistical significance of SP-A2 T2C miRNome expression levels compared to SP-A KO under FA or O_3_ exposure for males and females. **(A)** Male SP-A2 mice compared to KO exposed to FA; **(B)** Female SP-A2 mice compared to KO after FA exposure; **(C)** Male SP-A2 mice compared to KO after O_3_ exposure; **(D)** Female SP-A2 mice compared to KO after O_3_ exposure.

The expression levels of the hTG SP-A1 and SP-A2 T2C miRNomes were determined in males and females that were exposed to FA or O_3_ and compared to the corresponding KO T2C. The miRNome levels are presented as volcano plots to show the fold change regulation differences between levels of miRNAs in hTG and KO mice, as well as their statistical significance (Figures 2, 3).

After FA exposure, which serves as control, in our experimental model, we observed in Figure 2A, a very tightly packed cluster of data points with few data points exceeding the cutoff for significance (Bonferroni corrected *p* < 0.0166), indicating that there are only a few differences between FA-exposed SP-A1 males and KO males. In Figure 2B, when the same comparison is made with female mice, we observed a very similar pattern to that of males, with only two miRNAs to exceed the significance threshold. Following O_3_ exposure the SP-A1 male mice (Figure 2C) show a very different picture. There are many more differences (both in magnitude and in significance) between O_3_ exposed SP-A1 and KO males. SP-A1 females on the other hand show minimal changes compared to females KO (Figures 2C,D, respectively).

When we compared the SP-A2 hTG males and females after FA and OxS we do not see any robust differences as those seen with the SP-A1 male hTG (Figures 3A–D). It is immediately obvious that the pattern seen after OxS for the SP-A1 males is unique (shaded area of Figure 2C compared to the rest of the panels of Figure 2 and all panels of Figure 3) in the volcano plots analysis. This indicates that in response to OxS the male T2C miRNome of the SP-A1 mice is more responsive compared to the rest of the hTGs and exhibits a higher number of changed miRNAs that reach the Bonferroni corrected significance threshold *p* < 0.0166 (compare shaded area).

### Oxidative Stress Has a Major Effect on the Male T2C miRNome

**Figure 4:**
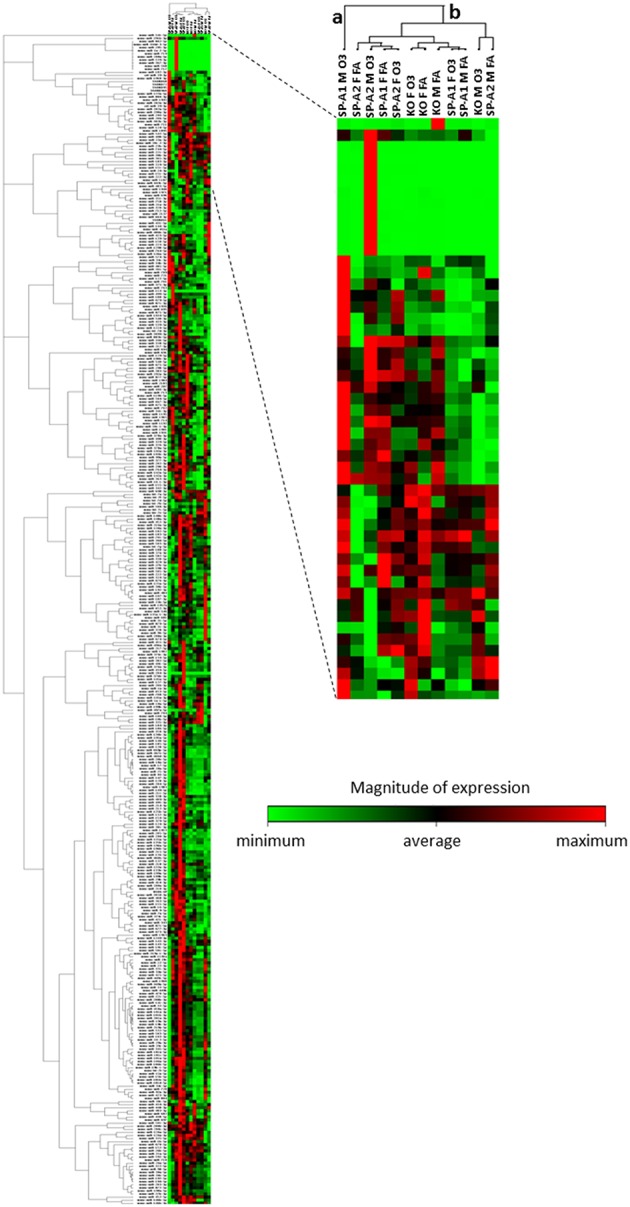
Heat map and dendrogram that shows a graphical representation of the fold regulation of miRNome expression between our different experimental groups and a non-supervised hierarchical clustering of these groups into clusters indicating co-regulated miRNAs across groups or individual samples. Cluster “a” consist only of the SP-A1M O_3_ group and cluster “b” contains all other groups studied. The colored bar indicates the magnitude of expression of each miRNA in each group.

We performed a non-supervised hierarchical clustering of our entire dataset to display a heat map with a dendrogram indicating co-regulated genes across groups or individual samples (Figure 4). We found two distinct clusters a and b, with clade a being the one of SP-A1 male T2C miRNome, while the rest of our experimental animals clustered together in group b. A two-way ANOVA test for sex and treatment effects showed that the F-stat for the SP-A1 mice regarding the sex effect is F = 161.91 with F crit = 3.84 and *p* = 2.79 × 10^−5^. The F-stat for the interaction between the two factors (sex and treatment) was F = 16.69 with F crit = 3.84 and *p* = 4.63 × 10^−5^. The same analysis for the SP-A2 mice miRNome did not show that sex, treatment, or the combination of these two factors were significantly different (F-stat for SP-A2 mice was F = 1.24 lower than the F crit = 3.84 and *p*-value not significant *p* = 0.265). These data show that in the T2C miRNome, there is a difference between sexes in response to O_3_ exposure as a function of SP-A variants.

### mRNA Targets of the Male SP-A1 T2C miRNome Associate With Cell Cycle, Apoptosis, and MAPK Pathway

To better understand and integrate the T2C miRNome data, we performed Ingenuity Pathway Analysis (IPA) for the T2C miRNAs whose expression was significantly altered by OxS. Only miRNAs that were shown to pass the corrected Bonferroni *p* < 0.0166 and a false discovery rate (FDR)-adjusted *q* < 0.05 were used to ensure that sex, treatment, gene, and array variability do not lead to false discoveries. Fifty-four miRNAs met the above criteria and were used for IPA. This analysis identified several mRNA transcripts, the expression of which could be affected by approximately half of the miRNAs selected for IPA. The targets identified include the following transcripts *Ago2, Ddx20, Plcg2, Irs1, Elf2, Jak2, Map2k4, Bcl2, Ccnd1*, and *Vhl* mRNAs. The levels of miRNAs that targeted the above molecules and were significantly changed in SP-A1 males in response to OxS are shown on Table 1.

**Table 1:** Levels and statistical significance of the male SP-A1 T2C miRNAs in OxS and shown by IPA to be directly associated with genes *Ago2, Ddx20, Plcg2, Irs1, Elf2, Bcl2, Jak2, Map2k4, Blc2, Ccnd1*, and *Vhl*.

**Mature miRNA ID**	**Fold regulation**	***p*-value**	**FDR**	**Target gene(s)**	**PMID**	Validation experiments*
			**(*q*-value)**			
miR-124-3p	−2.6902	0.006674	0.038944405	*Ago2, Ccnd1, Eif2*	27577603	HITS-CLIP
miR-135a-5p	−2.9089	0.001569	0.037656	*Jak2*	30854107	Luciferase
miR-141-3p	−3.3007	0.007623	0.038944405	*Map2k4*	28454307	WB, IHC, qRT-PCR
miR-143-3p	−3.4508	0.000843	0.029428364	*Bcl2*	29581736	WB
miR-143-5p	−2.5534	0.00237	0.038944405	*Bcl2*	20878132	qRT-PCR
miR-148a-3p	−2.193	0.00597	0.038944405	*Ppara* and indirectly *Bcl2*	26001136	Luciferase
miR-153-3p	−4.1674	0.000733	0.0281472	*Bcl2*	30537994	Luciferase
miR-190a-5p	−3.1766	0.003205	0.038944405	*Ddx20*		*in silico* report
miR-19b-3p	−3.5226	0.004329	0.038944405	*Ccnd1*	29455644	WB
miR-204-3p	2.2073	0.007978	0.038944405	N/A		
miR-208a-3p	−3.7777	0.004875	0.038944405	*Ddx20*		*in silico* report
miR-20b-5p	−2.2419	0.00638	0.038944405	Indirect effect on *Bcl2*	30816540	Luciferase
miR-219a-5p	−16.1578	0.000011	0.002112	*Plcg2*	20956612	qRT-PCR
miR-223-3p	6.3634	0.003918	0.038944405	*Irs1*	29286159	qRT-PCR
miR-26a-5p	−2.3634	0.006167	0.038944405	Indirect effect on *Htr1a*	30766477	Luciferase
miR-29b-3p	−2.6192	0.004297	0.038944405	*Ago2*		IP
miR-301a-3p	−3.0775	0.010054	0.045186977	Indirect effect in *Ago2*	28332581	qRT-PCR
miR-302a-3p	2.2073	0.007978	0.038944405	Indirect effect in Ccd1	28510621	Luciferase
miR-302a-5p	2.2073	0.007978	0.038944405	Indirect effect in Ccd1	28510621	Luciferase
miR-34b-3p	2.1409	0.004946	0.038944405	N/A		
miR-499-5p	−2.6842	0.000025	0.0032	*Sox6*	31076992	qRT-PCR
miR-539-3p	2.2073	0.007978	0.038944405	*Ntrk3*	21143953	Luciferase
miR-708-5p	−2.3546	0.008364	0.039182049	*Vhl*	21852381	qRT-PCR
miR-758-3p	3.3301	0.001181	0.032393143	Indirectly *Bcl2*	31138034	qRT-PCR

PMID, unique identifier number used in PubMed for miRNA; –, indicates downregulation; FDR, false discovery rate; HITS-CLIP, high-throughput sequencing of RNA isolated by cross-linking immunoprecipitation; WB, western blot; IHC, immunohistochemistry; qRT-PCR, quantitative reverse transcription polymerase chain reaction; IP, immunoprecipitation; N/A, non-applied; ^*^, last column shows the validation techniques used for each miRNA as noted in the PMID.

**Figure 5:**
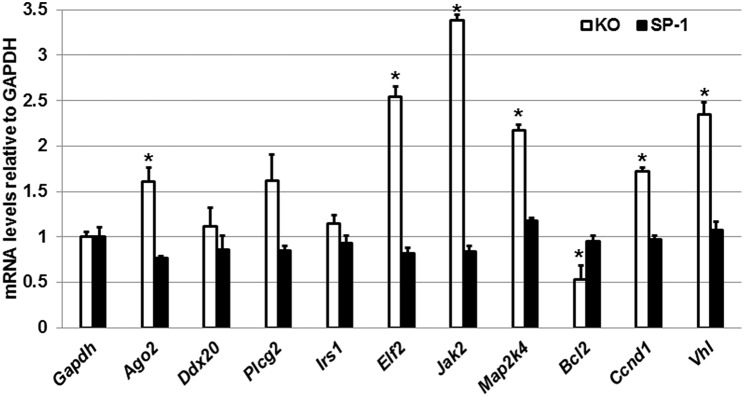
Effect of OxS in the T2C transcriptome of males. mRNA levels of *Gapdh, Ago2, Ddx20, Plcg2, Irs1, Elf2, Bcl2, Jak2, Map2k4, Blc2, Ccnd1*, and *Vhl* genes shown to be targeted by the T2C miRNome were measured in KO and SP-A1 male mice 4 h post O_3_ exposure. mRNA levels were measured by qRT-PCR and normalized to *Gapdh*. In KO the *Jak2, Elf2, Vhl, Mapk2, Ccnd1*, and *Ago2* were significantly upregulated by 3.4-, 2.5-, 2.3-, 2.2-, 1.7-, and 1.7- fold (*p* < 0.05), respectively, while the *Bcl2* was significantly downregulated by 0.5-fold. The levels of those mRNAs did not change in the SP-A1 mice. *means *p* < 0.05.

Next, we performed qRT-PCR to assess the expression levels of *Ago2, Ddx20, Plcg2, Irs1, Elf2, Jak2, Map2k4, Bcl2, Ccnd1*, and *Vhl* genes in the male KO and SP-A1 T2C (Figure 5). To our surprise, we observed that in response to OxS the levels of *Ago2, Elf2, Jak2, Map2k4, Ccnd1*, and *Vhl* were significantly upregulated in the KO T2C but remained unaffected in the SP-A1 T2C. Also, the *Bcl2* gene was significantly downregulated only in the KO T2C. The above molecules are involved in several pathways including mitogen activated protein kinases (*MAPK*) signaling pathway, cell cycle, and apoptosis.

### Effect of Gonadectomy and OxS on the Expression of miRNAs in SP-A1 Male and Female Mice

To study the effect of sex hormones on the expression of miRNAs after OxS, we performed miRNA expression analysis in T2C from gonadectomized (Gx) SP-A1 and KO male and female mice and compared it with that of non-gonadectomized (NGx) mice after O_3_ exposure.

**Figure 6:**
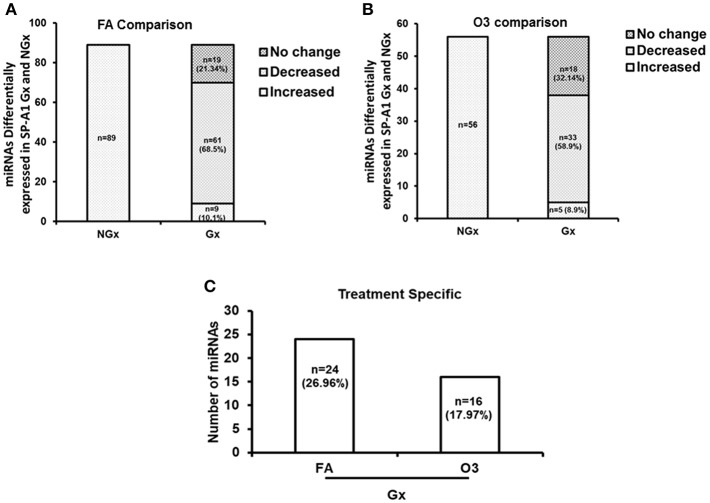
The effect of gonadectomy and OxS on T2C miRNA expression profiles of SP-A1 mice. **(A)** The differentially expressed miRNAs in SP-A1 non-gonadectomized (NGx) and gonadectomized (Gx) mice were identified after normalizing to corresponding NGx and Gx KO. NGx shows the miRNAs (*n* = 89) that changed significantly (≥2-fold) in FA when males were compared to females. Gx depicts the comparison of Gx values (male vs. female) to NGx (male vs. female). Out of the same 89 miRNAs (found to have their levels increased in NGx), 9 miRNAs (10.1%) showed a significant increase (≥2-fold) and 61 miRNAs (68.53%) showed a significant decrease (≥2-fold). **(B)** NGx shows the miRNAs (*n* = 56) that changed significantly (≥2-fold) in OxS when males were compared to females. Gx depicts the comparison of Gx values (male vs. female) to NGx (male vs. female). Out of the same 56 miRNAs (found to have their levels increased in NGx), 5 miRNAs (8.9%) showed a significant increase (≥2-fold) and 33 miRNAs (58.9%) showed a significant decrease (≥2-fold). **(C)** Depicts the comparison of the 89 and 56 differentially expressed miRNAs identified between Gx males and females. Out of 89 miRNAs studied, 24 miRNAs (26.96%) are significantly increased in FA (≥2-fold), and 16 miRNAs (17.97%) are significantly increased in OxS (≥2-fold).

For this analysis, we used 120 miRNAs that were identified in both NGx (males and females) and Gx (males and females) groups. Of these, in the NGx (males vs. females) group, 89 miRNAs had their levels significantly changed (fold change ≥2) after FA exposure (Figure 6A). In the Gx group (male vs. female) compared to the corresponding NGx (male vs. female) group, expression of 9 miRNAs (10.1%) was significantly increased (fold change ≥2), and expression of 61 miRNAs (68.53%) was significantly decreased (fold change ≥2) (Figure 6A). In response to OxS, the level of 56 miRNAs was significantly altered (≥2 fold) in NGx (male vs. female) groups (Figure 6B). Following, comparison of the Gx group (male vs. female) to the corresponding NGx (male vs. female) group, the expression of 5 miRNAs (8.9%) was significantly increased (≥2 fold), and the expression levels of 33 (58.9%) miRNAs was significantly decreased (≥2 fold) (Figure 6B). Of the 89 (Figure 6A) and 56 (Figure 6B) miRNAs differentially expressed in Gx males vs. females after FA and O_3_ exposure, 24 miRNAs (26.96%) are specific to FA exposure and 16 miRNAs (17.97%) are specific to O_3_ exposure (Figure 6C). Moreover, a one-way ANOVA pertaining to the gonadectomy effect on the miRNA expression showed a significant difference with F stat = 120.5 with F crit = 3.88 and *p* = 5.95 × 10^−23^ (see Supplementary File 1) indicating that sex hormones play a role.

**Figure 7:**
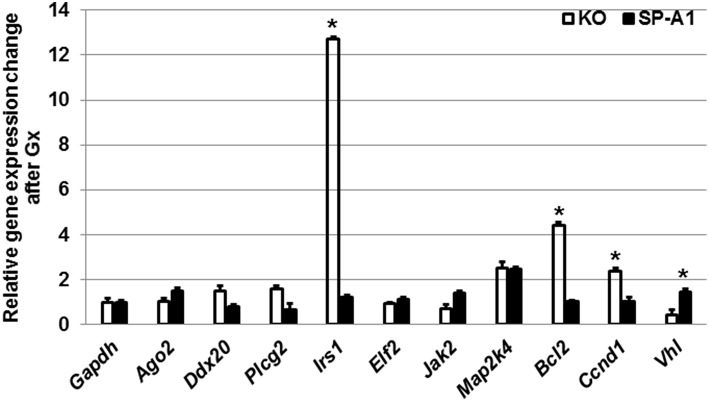
The effect of gonadectomy and OxS on genes that are targeted by the T2C miRNome. Genes were measured by qRT-PCR in KO and SP-A1 gonadectomized (Gx) male mice 4 h post O_3_ exposure and normalized to housekeeping gene *Gapdh*. The graph shows the relative expression changes of mRNAs Gx mice compared to non-gonadectomized. In the Gx KO the *Irs1, Bcl2*, and *Ccnd1* expression levels were increased 13-, 4.5-, and 2.8-fold (*p* < 0.05), respectively. In the Gx SP-A1 the *Vhl* gene was significantly increased in SP-A1 by 1.8-fold. *means *p* < 0.05.

Furthermore, we monitored the expression levels of the target genes discussed above after gonadectomy in both SP-A1 and KO mice (Figure 7). We found that Gx had a major effect in three genes (*Irs1, Bcl2, Vhl*) in KO mice. *Irs1* was upregulated in Gx KO only. No significant change was observed for IRS1 in the NGx KO (Figure 4). *Bcl2* and *Vhl* showed decrease and increase expression, respectively, compared to control *Gapdh* mRNA (Figure 7); both of these had shown the reverse in NGx (Figure 5). Of interest, *Ccnd1* was upregulated in KO regardless of Gx. These data indicate that sex hormones play an important role in the observed miRNA sex differences by affecting regulation of miRNAs, as most of the miRNAs that were increased in NGx were decreased in Gx.

## Discussion

We have previously shown a differential effect of SP-A1 and SP-A2 proteins on AM function (31, 37, 38), AM proteome (34, 35) and AM miRNome (42) as well as sex differences after OxS. In the present study, we investigated the effect of OxS on mouse T2C under the influence of either SP-A1 or SP-A2 and compared it to KO mice as well as studied the role of sex hormones in the T2C miRNome of the SP-A1 hTG mice. We found significant changes after O_3_ exposure in SP-A1 males but not in the other animals. When a non-supervised hierarchical clustering analysis on the entire dataset was performed, we observed that the O_3_-exposed SP-A1 male miRNome clustered separately from the rest of the experimental animals showing that OxS has a major effect on the male SP-A1 T2C miRNome. Also, a two-way ANOVA analysis showed that there is an interaction between the male sex hormones and the SP-A1 gene under the effect of OxS. Gonadectomy had a major effect on the expression of the T2C miRNome compared to non-gonadectomized mice. Our miRNome analysis in the T2C that was subjected to OxS was based on strong validation methodologies. Ingenuity Pathway Analysis (IPA), pairs miRNAs/mRNA targets based not only on the gold standard *in silico* predicted algorithms (miRBase, miRTarBase, miRWalk, Targetscan, etc.) but also on experimental data from the published literature. With IPA we showed that miRNAs that were changed significantly >2-fold in male SP-A1 T2C mice, targeted genes that are involved in the MAPK signaling pathway, cell cycle, and anti-apoptosis. We monitored/validated experimentally via qRT-PCR which of the predicted mRNA targets are responding to the effect of OxS. Gene expression analysis of the target mRNAs of interest surprisingly showed that the OxS affected predominantly the KO mouse while the SP-A1 mouse showed no significant shifts in the expression levels of the same genes. Gonadectomy of male SP-A1 and KO mice prior to O_3_ exposure led to significant changes in the expression levels of three genes (*Irs1, Bcl2*, and *Vhl*) in KO, whereas the expression of *Ccnd1* remained increased in KO regardless of Gx. These data indicate that sex differences are in part attributable to circulating gonadal hormones (51), which are believed to influence the innate immune responses. The specific roles of these hormones and the underlying mechanisms of regulation remain yet to be explored.

The mRNA targets of the SP-A1 T2C miRNAs that were changed significantly in the non-gonadectomized males under OxS included genes being involved in the *MAPK* signaling, apoptosis, and cell cycle. Although the activation of the *MAPK* pathway by OxS has been described before in other systems and tissues (55–57), in the present study our data show that there are miRNAs that may regulate genes of the *MAPK* signaling pathways in the respiratory alveolar epithelial T2C. The validation experiments on the mRNA targets showed that the mRNAs of interest (that were targeted by significantly changed miRNAs, Figure 2C) are particularly responsive in the male KO but not responsive in SP-A1 except the *Bcl2* which showed increased levels in SP-A1. The increase of *Bcl2* in SP-A1 indicates that the SP-A1 T2C may be protected against apoptosis. Previously, we have shown that AM of SP-A2 hTG mice were also protected from apoptosis under OxS (42), indicating a differential effect of the two SP-A genes, *SFTPA1* and *SFTPA2*, in T2C and AM, respectively, with regards to apoptosis. However, the fact that most of the target genes in SP-A1 remained unaffected after OxS is puzzling. We speculate that this may in part be due to: (1) The time point used (4 h after OxS) for study is not optimal to assess mRNA levels of target genes in SP-A1; (2) the target genes may recover from OxS faster in SP-A1 than in KO; (3) SP-A1 may protect other molecules from harmful effects of OxS by being more readily oxidized by scavenging ROS (48); (4) SP-A1 may protect the T2C by affecting functions modulated by molecules that are not regulated by miRNAs. An example of this may be its role in surfactant structural organization (41) and potentially lung function. Another interesting observation was the significant upregulation of Von Hippel-Lindau (*Vhl*) mRNA, targeted by miR-708-5p (58), in the gonadectomized (Gx) SP-A1 T2C when the experimental male mice were exposed to O_3_. It is known that *Vhl* is part of a degradation complex that removes damaged or unnecessary proteins and helps maintain the normal functions of cells (59). In particular, this degradation complex is known to degrade proteins when oxygen levels are lower than normal, such as hypoxia (60) and possibly in OxS. The increase in *Vhl* in Gx SP-A1 mice (but not in NGx) indicates that sex hormones may play a role in its expression in SP-A1 mice.

The increase of several targets in KO but not in SP-A1 may point to deficiencies in KO and that these increases may reflect a more active gene regulation to overcome the effects of OxS. These include, the mitogen-Activated Protein Kinase 4 (*Map2k4*) and Janus Kinase 2 (*Jak2*), which are targeted by miR-141-3p (61) and miR-135a-5p (62) and shown here to change by >2-fold; both targets are involved in the *MAPK* signaling. These could transmit the OxS distress signal across the cell membrane to the DNA in the nucleus triggering a number of different functions such as apoptosis, cell differentiation and proliferation. The argonaute 2 (*Ago2*) mRNA, which is targeted by miR-124-3p and miR-29b-3p (63), was increased significantly in the KO. *Ago2* is an important enzyme in the biogenesis of miRNAs that plays a role in the formation of the RNA-induced silencing complex (64). Of interest, cyclin D1 (*Ccnd1*) was upregulated in Gx and NGx KO males indicating independence of circulating hormones, and possibly a dependence on the presence or absence of SP-A, no change in *Ccnd1* was observed in SP-A1 T2C either in Gx or NGx males. The eukaryotic initiation factor 2 (*eIF2*) was upregulated in NGx KO but not in the Gx KO mice indicating a role of hormones in its expression. These mRNAs (*Ccnd1* and *elF2*) are targeted by miR-124-3p. This molecule is known to regulate global and specific mRNA translation in response to stress-related signals (such as OxS) (65).

**Figure 8:**
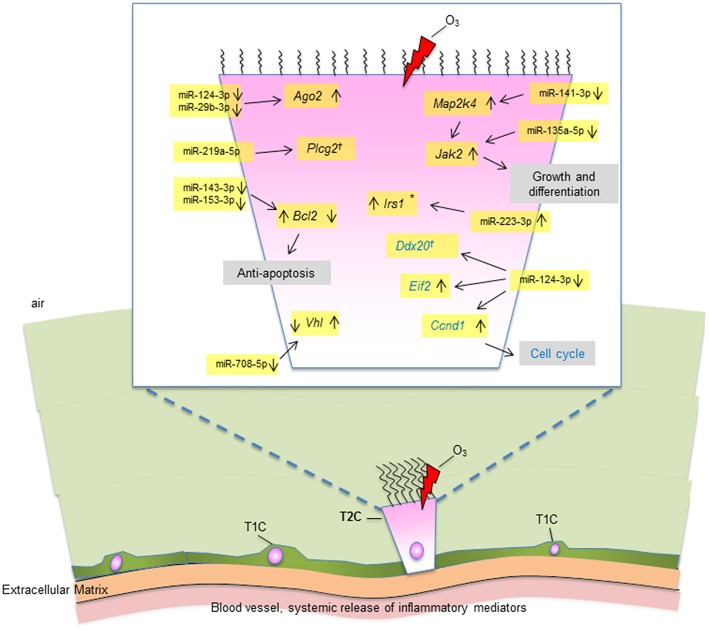
A diagrammatic representation of miRNAs and their targets in T2C under OxS. Targets with significantly changed levels in KO compared to SP-A1 are members of several pathways as shown in figure. These include *MAPK* and *JAK2* signaling pathways, cell cycle, anti-apoptosis, and other. The miRNAs and the mRNA levels of their target genes studied in the present study are highlighted in yellow. Up (↑) or down (↓) on the right of the target gene indicate increase or decrease in KO in non-gondectomized (NGx) mice. Up (↑) or down (↓) on the left of the target gene indicate increase or decrease in gondectomized (Gx) mice. The expression of 3 genes (*Irs1, Bcl2, And Vhl*) changed after gonadectomy: *Bcl2* and *Vhl*, respectively showed increase and decrease in KO, which is the reverse of what is seen in the NGx KO. *Irs1* increased in Gx KO only, no change in NGx (*). The expression of *Ccnd1* was increased in both NGx and Gx KO indicating hormone-independent expression. The expression of *Plcg2* and *Ddx20* did not change significantly after OxS in either NGx or GX KO (†).

Collectively, our data show that OxS has a major effect on the male SP-A1 T2C miRNome. The targets of the significant miRNAs are implicated in several pathways that include the *MAPK* signaling pathway (*Mapk, Jak2, Irs1*), cell cycle (*Ccnd1*), anti-apoptosis (*Bcl2*), protein degradation (*Vhl*), and other. These observations diagrammatically are depicted in Figure 8. The limitations of the present study are that we studied a single time point and we did not look at the protein levels of the targeted mRNAs. Our publication on the alveolar macrophages (AM) miRNome showed that in response to OxS, it was SP-A2 that had a major effect on the male AM with pro-inflammatory, anti-apoptotic, and anti-oxidant pathways playing a role (42). The two studies together indicate that there is a differential role of SP-A1 and SP-A2 in the alveolar cells. The SP-A1 findings in the present study are consistent with our previous observations where SP-A1 is shown to play a role in the surfactant structural organization (41), and may also play an important role on the integrity and function of T2C. SP-A2 on the other hand has been shown to not only affect the AM male miRNome but also exhibit a higher innate immune activity (31, 36, 38).

We conclude that dysregulation of either SP-A1 or SP-A2 may affect the innate immunity and/or surfactant structure and potentially lung function. Both processes are essential for normal lung function and derangement of the regulation of either gene may be a problem in pulmonary diseases, including OxS.

## Ethics Statement

All protocols used in this study were evaluated and approved by the Pennsylvania State University College of Medicine Institutional Animal Care and Use Committee and conformed to the guidelines of the National Institutes of Health on the care and use of laboratory animals.

## Author Contributions

GN performed experiments, run statistics, analyzed and synthesized the data, contributed to the manuscript writing. NT performed experiments, analyzed and synthesized the data, contributed to the manuscript writing. XZ and TU performed maintenance and breeding of mouse lines, exposed mice to experimental conditions (FA & O_3_) and isolated alveolar type II cells. FD performed all gonadectomy experiments. DP contributed to data analysis and synthesis and manuscript writing. JF designed and provided oversight to the entire project, involved in data analysis, integration, and writing of the manuscript.

### Conflict of Interest Statement

The authors declare that the research was conducted in the absence of any commercial or financial relationships that could be construed as a potential conflict of interest.

## Abbreviations

*Ago2*: argonaute 2
AM: alveolar macrophages
*Bcl2*: B-cell lymphoma 2
Ccnd1: cyclin D1
COPD: chronic obstructive lung disease
CRS: chronic rhinosinusitis
*Ddx20*: dead-box helicase 20
eIF2: eukaryotic initiation factor 2
FA: filtered air
Gx: gonadectomized
FDR: false discovery rate
hTG: humanized transgenic
*Irs1*: Insulin Receptor Substrate 1
*Jak2*: Janus Kinase 2
KO: SP-A knock-out
*Map2k4*: Mitogen-Activated Protein Kinase 4
*MAPK*: mitogen-activated protein kinases
miRNAs: microRNAs
NGx: non-gonadectomized
O3: ozone
OxS: oxidative stress
*Plcg2*: Phospholipase C Gamma 2
ROS: reactive oxygen species
*SFTPA1*: gene encoding SP-A1
*SFTPA2*: gene encoding SP-A2
SP-A: surfactant protein A
SP-B: surfactant protein B
SP-C: surfactant protein C
SP-D: surfactant protein D
T1C: alveolar type I cells
T2C: alveolar type II cells
*Vhl*: Von Hippel-Lindau protein.

## References

[1] Fordham MT Mulligan JK Casey SE Mulligan RM Wang EW Sansoni ER. Reactive oxygen species in chronic rhinosinusitis and secondhand smoke exposure. Otolaryngol Head Neck Surg. (2013) 149:633–8. 10.1177/0194599813496377 23838308

[2] Al-Hegelan M Tighe RM Castillo C Hollingsworth JW. Ambient ozone and pulmonary innate immunity. Immunol Res. (2011) 49:173–91. 10.1007/s12026-010-8180-z 21132467 PMC3747041

[3] Holguin F. Oxidative stress in airway diseases. Ann Am Thorac Soc. (2013) 10(Suppl.):S150–7. 10.1513/AnnalsATS.201305-116AW 24313766

[4] Whitsett JA Alenghat T. Respiratory epithelial cells orchestrate pulmonary innate immunity. Nat Immunol. (2015) 16:27–35. 10.1038/ni.3045 25521682 PMC4318521

[5] Meyer M Jaspers I. Respiratory protease/antiprotease balance determines susceptibility to viral infection and can be modified by nutritional antioxidants. Am J Physiol Lung Cell Mol Physiol. (2015) 308:L1189–201. 10.1152/ajplung.00028.2015 25888573 PMC4587599

[6] Voter KZ Whitin JC Torres A Morrow PE Cox C Tsai Y. Ozone exposure and the production of reactive oxygen species by bronchoalveolar cells in humans. Inhal Toxicol. (2001) 13:465–83. 10.1080/08958370151131837 11445887

[7] Yang L Chen X Simet SM Hu G Cai Y Niu F. Reactive oxygen species/hypoxia-inducible factor-1α/platelet-derived growth factor-BB autocrine loop contributes to cocaine-mediated alveolar epithelial barrier damage. Am J Respir Cell Mol Biol. (2016) 55:736–48. 10.1165/rcmb.2016-0096OC 27391108 PMC5105185

[8] Finlayson-pitts BJ Mautz WJ Lai CC Bufalino C Messer K Mestas J Are changes in breathing pattern on exposure to ozone related to changes in pulmonary surfactant? Inhal Toxicol. (1994) 6:267–87. 10.3109/08958379408995236

[9] Chambers RC Mercer PF. Mechanisms of alveolar epithelial injury, repair, and fibrosis. Ann Am Thorac Soc. (2015) 12(Suppl 1.):S16–20. 10.1513/AnnalsATS.201410-448MG 25830828 PMC4430974

[10] Schneeberger E Alveolar type I cells. In: Crystal RG West JB Weibel ER Barnes PJ editors. The Lung:Scientific Foundations. 2nd Edn Philadelphia, PA: Lippincott-Raven (1997). 535–42.

[11] Crosby LM Waters CM. Epithelial repair mechanisms in the lung. Am J Physiol Lung Cell Mol Physiol. (2010) 298:L715–31. 10.1152/ajplung.00361.2009 20363851 PMC2886606

[12] Ghosh MC Gorantla V Makena PS Luellen C Sinclair SE Schwingshackl A. Insulin-like growth factor-I stimulates differentiation of ATII cells to ATI-like cells through activation of Wnt5a. Am J Physiol Lung Cell Mol Physiol. (2013) 305:L222–8. 10.1152/ajplung.00014.2013 23709620 PMC3743013

[13] Griese M. Pulmonary surfactant in health and human lung diseases: state of the art. Eur Respir J. (1999) 13:1455–76. 10.1183/09031936.99.13614779 10445627

[14] Crouch E Wright JR. Surfactant proteins a and d and pulmonary host defense. Annu Rev Physiol. (2001) 63:521–54. 10.1146/annurev.physiol.63.1.521 11181966

[15] Phelps DS. Surfactant regulation of host defense function in the lung: a question of balance. Pediatr Pathol Mol Med. (2001) 20:269–92. 10.1080/15513810109168822 11486734

[16] Wright JR. Immunoregulatory functions of surfactant proteins. Nat Rev Immunol. (2005) 5:58–68. 10.1038/nri1528 15630429

[17] Floros J Wang G Mikerov AN. Genetic complexity of the human innate host defense molecules, surfactant protein A1 (SP-A1) and SP-A2–impact on function. Crit Rev Eukaryot Gene Exp. (2009) 19:125–37. 10.1615/CritRevEukarGeneExpr.v19.i2.30 19392648 PMC2967201

[18] LeVine AM Bruno MD Huelsman KM Ross GF Whitsett JA Korfhagen TR. Surfactant protein A-deficient mice are susceptible to group B streptococcal infection. J Immunol. (1997) 158:4336–40. 9126996

[19] LeVine AM Kurak KE Bruno MD Stark JM Whitsett JA Korfhagen TR. Surfactant protein-A-deficient mice are susceptible to *Pseudomonas aeruginosa* infection. Am J Respir Cell Mol Biol. (1998) 19:700–8. 10.1165/ajrcmb.19.4.3254 9761768

[20] LeVine AM Whitsett JA Gwozdz JA Richardson TR Fisher JH Burhans MS. Distinct effects of surfactant protein A or D deficiency during bacterial infection on the lung. J Immunol. (2000) 165:3934–40. 10.4049/jimmunol.165.7.3934 11034401

[21] Mikerov AN Haque R Gan X Guo X Phelps DS Floros J. Ablation of SP-A has a negative impact on the susceptibility of mice to Klebsiella pneumoniae infection after ozone exposure: sex differences. Respir Res. (2008) 9:77. 10.1186/1465-9921-9-77 19055785 PMC2655296

[22] Mikerov AN Hu S Durrani F Gan X Wang G Umstead TM. Impact of sex and ozone exposure on the course of pneumonia in wild type and SP-A (-/-) mice. Microb Pathog. (2012) 52:239–49. 10.1016/j.micpath.2012.01.005 22285567 PMC3608432

[23] Mikerov AN Gan X Umstead TM Miller L Chinchilli VM Phelps DS. Sex differences in the impact of ozone on survival and alveolar macrophage function of mice after Klebsiella pneumoniae infection. Respir Res. (2008) 9:24. 10.1186/1465-9921-9-24 18307797 PMC2268931

[24] Phelps DS Umstead TM Quintero OA Yengo CM Floros J. *In vivo* rescue of alveolar macrophages from SP-A knockout mice with exogenous SP-A nearly restores a wild type intracellular proteome; actin involvement. Proteome Sci. (2011) 9:67. 10.1186/1477-5956-9-67 22035134 PMC3219558

[25] Phelps DS Umstead TM Floros J. Sex differences in the response of the alveolar macrophage proteome to treatment with exogenous surfactant protein-A. Proteome Sci. (2012) 10:44. 10.1186/1477-5956-10-44 22824420 PMC3570446

[26] Tsotakos N Phelps DS Yengo CM Chinchilli VM Floros J. Single-cell analysis reveals differential regulation of the alveolar macrophage actin cytoskeleton by surfactant proteins A1 and A2: implications of sex and aging. Biol Sex Differ. (2016) 7:18. 10.1186/s13293-016-0071-0 26998217 PMC4797174

[27] Kehrl HR Vincent LM Kowalsky RJ Horstman DH O'Neil JJ McCartney WH. Ozone exposure increases respiratory epithelial permeability in humans. Am Rev Respir Dis. (1987) 135:1124–8. 3579012 10.1164/arrd.1987.135.5.1124

[28] Huang W Wang G Phelps DS Al-Mondhiry H Floros J. Human SP-A genetic variants and bleomycin-induced cytokine production by THP-1 cells: effect of ozone-induced SP-A oxidation. Am J Physiol Lung Cell Mol Physiol. (2004) 286:L546–53. 10.1152/ajplung.00267.2003 14617519

[29] Janic B Umstead TM Phelps DS Floros J. Modulatory effects of ozone on THP-1 cells in response to SP-A stimulation. Am J Physiol Lung Cell Mol Physiol. (2005) 288:L317–25. 10.1152/ajplung.00125.2004 15466251

[30] Cho HY Morgan DL Bauer AK Kleeberger SR. Signal transduction pathways of tumor necrosis factor–mediated lung injury induced by ozone in mice. Am J Respir Crit Care Med. (2007) 175:829–39. 10.1164/rccm.200509-1527OC 17255564 PMC1899292

[31] Mikerov AN Umstead TM Gan X Huang W Guo X Wang G. Impact of ozone exposure on the phagocytic activity of human surfactant protein A (SP-A) and SP-A variants. Am J Physiol Lung Cell Mol Physiol. (2008) 294:L121–30. 10.1152/ajplung.00288.2007 17981957 PMC2964667

[32] Haque R Umstead TM Freeman WM Floros J Phelps DS. The impact of surfactant protein-A on ozone-induced changes in the mouse bronchoalveolar lavage proteome. Proteome Sci. (2009) 7:12. 10.1186/1477-5956-7-12 19323824 PMC2666657

[33] Connor AJ Laskin JD Laskin DL. Ozone-induced lung injury and sterile inflammation. Role of toll-like receptor 4. Exp Mol Pathol. (2012) 92:229–35. 10.1016/j.yexmp.2012.01.004 22300504 PMC3507381

[34] Phelps DS Umstead TM Silveyra P Hu S Wang G Floros J. Differences in the alveolar macrophage proteome in transgenic mice expressing human SP-A1 and SP-A2. J Proteom Genom Res. (2013) 1:2–26. 10.14302/issn.2326-0793.jpgr-12-207 24729982 PMC3981560

[35] Phelps DS Umstead TM Floros J. Sex differences in the acute *in vivo* effects of different human SP-A variants on the mouse alveolar macrophage proteome. J Proteomics. (2014) 108:427–44. 10.1016/j.jprot.2014.06.007 24954098 PMC4128237

[36] Wang G Umstead T Phelps D Al-Mondhiry H Floros J. The effect of ozone exposure on the ability of human surfactant protein a variants to stimulate cytokine production. Environ Health Perspect. (2002) 110:79–84. 10.1289/ehp.0211079 11781168 PMC1240696

[37] Mikerov AN Umstead TM Huang W Liu W Phelps DS Floros J. SP-A1 and SP-A2 variants differentially enhance association of *Pseudomonas aeruginosa* with rat alveolar macrophages. Am J Physiol Lung Cell Mol Physiol. (2005) 288:L150–8. 10.1152/ajplung.00135.2004 15377498

[38] Mikerov A Wang G Umstead T Zacharatos M Thomas N Phelps D. Surfactant protein A2 (SP-A2) variants expressed in CHO cells stimulate phagocytosis of *Pseudomonas aeruginosa* more than do SP-A1 variants. Infect Immun. (2007) 75:1403–12. 10.1128/IAI.01341-06 17220308 PMC1828577

[39] Wang G Bates-Kenney SR Tao JQ Phelps DS Floros J. Differences in biochemical properties and in biological function between human SP-A1 and SP-A2 variants, and the impact of ozone-induced oxidation. Biochemistry. (2004) 43:4227–39. 10.1021/bi036023i 15065867

[40] Wang G Taneva S Keough KM Floros J. Differential effects of human SP-A1 and SP-A2 variants on phospholipid monolayers containing surfactant protein B. Biochim Biophys Acta. (2007) 1768:2060–9. 10.1016/j.bbamem.2007.06.025 17678872 PMC2964661

[41] Lopez-Rodriguez E Pascual A Arroyo R Floros J Perez-Gil J. Human pulmonary surfactant protein SP-A1 provides maximal efficiency of lung interfacial films. Biophys J. (2016) 111:524–36. 10.1016/j.bpj.2016.06.025 27508436 PMC4982931

[42] Noutsios GT Thorenoor N Zhang X Phelps DS Umstead TM Durrani F. SP-A2 contributes to miRNA-mediated sex differences in response to oxidative stress: pro-inflammatory, anti-apoptotic, and anti-oxidant pathways are involved. Biol Sex Differ. (2017) 8:37. 10.1186/s13293-017-0158-2 29202868 PMC5716385

[43] Thorenoor N Zhang X Umstead TM Scott Halstead E Phelps DS Floros J. Differential effects of innate immune variants of surfactant protein-A1 (SFTPA1) and SP-A2 (SFTPA2) in airway function after *Klebsiella pneumoniae* infection and sex differences. Respir Res. (2018) 19:23. 10.1186/s12931-018-0723-1 29394894 PMC5797374

[44] Thorenoor N Umstead T Zhang X Phelps D Floros J. Survival of surfactant protein-A1 and SP-A2 transgenic mice after Kle*bsiella pneumoniae in*fection, exhibits sex-, gene-, and variant specific differences; treatment with surfactant protein improves survival. Front Immunol. (2018) 9:2404. 10.3389/fimmu.2018.02404 30459763 PMC6232836

[45] Silveyra P Wang G Floros J. Human SP-A1 (SFTPA1) variant-specific 3' UTRs and poly(A) tail differentially affect the in vitro translation of a reporter gene. Am J Physiol Lung Cell Mol Physiol. (2010) 299:L523–34. 10.1152/ajplung.00113.2010 20693318 PMC2957414

[46] Silveyra P Chroneos ZC DiAngelo SL Thomas NJ Noutsios GT Tsotakos N. Knockdown of Drosha in human alveolar type II cells alters expression of SP-A in culture: a pilot study. Exp Lung Res. (2014) 40:354–66. 10.3109/01902148.2014.929757 25058539 PMC4197128

[47] Wang G Guo X Diangelo S Thomas NJ Floros J. Humanized SFTPA1 and SFTPA2 transgenic mice reveal functional divergence of SP-A1 and SP-A2: formation of tubular myelin *in vivo* requires both gene products. J Biol Chem. (2010) 285:11998–2010. 10.1074/jbc.M109.046243 20048345 PMC2852938

[48] Haque R Umstead TM Ponnuru P Guo X Hawgood S Phelps DS. Role of surfactant protein-A (SP-A) in lung injury in response to acute ozone exposure of SP-A deficient mice. Toxicol Appl Pharmacol. (2007) 220:72–82. 10.1016/j.taap.2006.12.017 17307210 PMC1906716

[49] Umstead TM Phelps DS Wang G Floros J Tarkington BK. *In vitro* exposure of proteins to ozone. Toxicol Mech Methods. (2002) 12:1–16. 10.1080/1537-650291895739 20597812

[50] Messier EM Mason RJ Kosmider B. Efficient and rapid isolation and purification of mouse alveolar type II epithelial cells. Exp Lung Res. (2012) 38:363–73. 10.3109/01902148.2012.713077 22888851

[51] Durrani F Phelps DS Weisz J Silveyra P Hu S Mikerov AN. Gonadal hormones and oxidative stress interaction differentially affects survival of male and female mice after lung *Klebsiella pneumoniae* infection. Exp Lung Res. (2012) 38:165–72. 10.3109/01902148.2011.654045 22394250 PMC3651915

[52] Sun J Nishiyama T Shimizu K Kadota K. TCC: an R package for comparing tag count data with robust normalization strategies. BMC Bioinformatics. (2013) 14:219. 10.1186/1471-2105-14-219 23837715 PMC3716788

[53] Desvignes T Batzel P Berezikov E Eilbeck K Eppig JT McAndrews MS. miRNA nomenclature: a view incorporating genetic origins, biosynthetic pathways, and sequence variants. Trends Genet. (2015) 31:613–26. 10.1016/j.tig.2015.09.002 26453491 PMC4639415

[54] Piletič K Kunej T. Minimal standards for reporting microRNA:target interactions. OMICS. (2017) 21:197–206. 10.1089/omi.2017.0023 28388300

[55] Gaitanaki C Konstantina S Chrysa S Beis I. Oxidative stress stimulates multiple MAPK signalling pathways and phosphorylation of the small HSP27 in the perfused amphibian heart. J Exp Biol. (2003) 206(Pt 16):2759–69. 10.1242/jeb.00483 12847121

[56] Matos TJ Duarte CB Gonçalo M Lopes MC. Role of oxidative stress in ERK and p38 MAPK activation induced by the chemical sensitizer DNFB in a fetal skin dendritic cell line. Immunol Cell Biol. (2005) 83:607–14. 10.1111/j.1440-1711.2005.01378.x 16266312

[57] Son Y Cheong YK Kim NH Chung HT Kang DG Pae HO. Mitogen-activated protein kinases and reactive oxygen species: how can ROS activate MAPK pathways? J Signal Transduct. (2011) 2011:792639. 10.1155/2011/792639 21637379 PMC3100083

[58] Li M Wang Y Song Y Bu R Yin B Fei X. MicroRNAs in renal cell carcinoma: a systematic review of clinical implications (Review). Oncol Rep. (2015) 33:1571–8. 10.3892/or.2015.3799 25682771 PMC4358077

[59] Iturrioz X Durgan J Calleja V Larijani B Okuda H Whelan R. The von Hippel-Lindau tumour-suppressor protein interaction with protein kinase Cdelta. Biochem J. (2006) 397:109–20. 10.1042/BJ20060354 16669786 PMC1479743

[60] Krek W. VHL takes HIF's breath away. Nat Cell Biol. (2000) 2:E121–3. 10.1038/35017129 10878820

[61] Ding L Yu LL Han N Zhang BT. miR-141 promotes colon cancer cell proliferation by inhibiting MAP2K4. Oncol Lett. (2017) 13:1665–71. 10.3892/ol.2017.5653 28454307 PMC5403415

[62] Navarro A Diaz T Martinez A Gaya A Pons A Gel B. Regulation of JAK2 by miR-135a: prognostic impact in classic Hodgkin lymphoma. Blood. (2009) 114:2945–51. 10.1182/blood-2009-02-204842 19666866

[63] Turchinovich A Burwinkel B. Distinct AGO1 and AGO2 associated miRNA profiles in human cells and blood plasma. RNA Biol. (2012) 9:1066–75. 10.4161/rna.21083 22858679 PMC3551861

[64] Macfarlane LA Murphy PR. MicroRNA: Biogenesis, function and role in cancer. Curr Genomics. (2010) 11:537–61. 10.2174/138920210793175895 21532838 PMC3048316

[65] Weeks A Agnihotri S Lymer J Chalil A Diaz R Isik S. Epithelial cell transforming 2 and aurora kinase B modulate formation of stress granule-containing transcripts from diverse cellular pathways in astrocytoma cells. Am J Pathol. (2016) 186:1674–87. 10.1016/j.ajpath.2016.02.013 27106762

